# Epidemiological Surveillance of Lyme Borreliosis in Bavaria, Germany, 2013–2020

**DOI:** 10.3390/microorganisms9091872

**Published:** 2021-09-03

**Authors:** Merle Margarete Böhmer, Katharina Ens, Stefanie Böhm, Susanne Heinzinger, Volker Fingerle

**Affiliations:** 1 Department for Infectious Disease Epidemiology, Taskforce Infectiology, Bavarian Health and Food Safety Authority, Lazarettstrasse 67, 80636 Munich, Germany; 2 Institute of Social Medicine and Health Systems Research, Otto-von-Guericke-University Magdeburg, 39120 Magdeburg, Germany; 3 Institute for Medical Information Processing, Biometry and Epidemiology (IBE), Pettenkofer School of Public Health, Ludwig-Maximilians-University Munich (LMU), 81377 Munich, Germany; katharina-ens@gmx.de; 4 Bavarian Health and Food Safety Authority, 80636 Munich, Germany; stefanie.boehm@lgl.bayern.de; 5 Bavarian Health and Food Safety Authority, 85764 Oberschleissheim, Germany; susanne.heinzinger@lgl.bayern.de (S.H.); volker.fingerle@lgl.bayern.de (V.F.); 6 National Reference Centre for Borrelia, 85764 Oberschleissheim, Germany

**Keywords:** Lyme borreliosis, Lyme disease, *Borrelia burgdorferi*, Bavaria, Germany, surveillance, epidemiology, incidence, regional distribution

## Abstract

Lyme borreliosis (LB) is the most common tick-borne disease in Germany. Mandatory notification of acute LB manifestations (erythema migrans (EM), neuroborreliosis (NB), and Lyme arthritis (LA)) was implemented in Bavaria on 1 March 2013. We aimed to describe the epidemiological situation and to identify LB risk areas and populations. Therefore, we analyzed LB cases notified from March 2013 to December 2020 and calculated incidence (cases/100,000 inhabitants) by time, place, and person. Overall, 35,458 cases were reported during the study period (EM: 96.7%; NB: 1.7%; LA: 1.8%). The average incidence was 34.3/100,000, but annual incidence varied substantially (2015: 23.2; 2020: 47.4). Marked regional differences at the district level were observed (annual average incidence range: 4–154/100,000). The Bavarian Forest and parts of Franconia were identified as high-risk regions. Additionally, high risk for LB was found in 5–9-year-old males and in 60–69-year-old females. The first group also had the highest risk of a severe disease course. We were able to identify areas and populations in Bavaria with an increased LB risk, thereby providing a basis for targeted measures to prevent LB. Since LB vaccination is currently not available, such measures should comprise (i) avoiding tick bites, (ii) removing ticks rapidly after a bite, and (iii) treating LB early/adequately.

## 1. Introduction

Lyme disease or Lyme borreliosis (LB) is a vector-borne disease caused by the spirochaete species of the *Borrelia (B.) burgdorferi* sensu lato complex. In Europe, five *B. burgdorferi* species (*B. burgdorferi* sensu stricto (s.s.), *B. afzelii, B. garinii, B. bavariensis*, and *B. spielmanii*) are present and assured to be pathogenic to humans [1]. In Western Europe, including Germany, *B. burgdorferi* is predominantly transmitted via the bite of the hard-bodied tick species *Ixodes ricinus* [1,2]. LB is the most common tick-borne disease in Germany, followed by tick-borne encephalitis (TBE), a viral disease. Germany is considered a high LB-prevalence country, with *B. burgdorferi* infection prevalence in ticks ranging from around 10% (nymphs) to >20% (adult ticks) [2,3]. Since the 1980s, Bavaria has been well known as an endemic area for LB. Based solely on focal data, *Ixodes ricinus* is present and is the dominant tick species in all suitable habitats. Tick density and the *Borrelia* infection rate may largely differ even between closely located areas, but the latter is on average around 1% for larvae, 10% for nymphs, and 20% for adult ticks [3,4,5,6,7]. Infection in humans is rather common. In two large population-based cross-sectional studies, the seroprevalence of antibodies against *B. burgdorferi* was found to be 4.0% in children and adolescents (<18 years) and 9.4% in adults (≥18 years) living in Germany, with case numbers being highest in southern Germany [8,9].

LB most commonly manifests as erythema migrans (EM), an expanding skin lesion or rash that occurs 3–30 days after a tick bite [10,11,12]. If not treated, the infection can disseminate into other tissues and organs, causing more severe disease manifestations [1,10]. The most common early disseminated form of LB is acute neuroborreliosis (NB). Clinical manifestations of NB include radiculopathy, meningitis, and cranial neuropathy (neuritis cranialis, most frequently presenting as facial palsy). Late disseminated forms of LB can occur months or even years after the initial infection and include acrodermatitis chronica atrophicans, as well as the most common late manifestation Lyme arthritis (LA) and rarely late NB [1,10,11,13,14]. The vast majority of LB patients, however, can be successfully treated with antibiotics [11,15,16].

Risk of infection depends on multiple factors including tick density, the proportion of infected ticks within a tick population, micro- and macroclimatic conditions, and the duration between tick bite and removal of the tick, as well as the individual immunity against *B. burgdorferi* of the person bitten. Disease severity additionally depends on the *B. burgdorferi* species causing the infection [2,17]. Seeing as a vaccine against LB is currently not available, personal protective measures such as wearing appropriate clothing and using repellents, as well as correct behavior after a tick bite (e.g., prompt removal of ticks, disinfection of bite wounds) are crucial for preventing infection [18,19].

LB is not a notifiable disease at the federal level in Germany. However, 9 of the 16 German federal states have introduced state-specific notification for LB. In the federal state of Bavaria (~13.1 million inhabitants; [20]) in southeast Germany, mandatory notification of LB was implemented on 1 March 2013. The rationale for the implementation was (i) to gain information on disease burden and epidemiology, (ii) to increase awareness of LB among physicians in Bavaria, and (iii) to monitor a possible climate change-related increase in LB [21]. In addition to the mandatory notification, a physician-based sentinel system (Lyme Disease Incidence Sentinel; LYDI) was implemented in Bavaria in mid-2012 in order to assess the quality of notification data and gain further information on the clinical symptoms and therapy of LB [22]. At the European level, distribution of NB has been monitored by the ECDC using a uniform case definition since 22 June 2018. For this purpose, data are collected via the epidemiological surveillance network comprising the European Commission, the ECDC, and the national public health authorities [23].

In our report, we present results of the first 8 years of LB surveillance in Bavaria, analyzing both notification and LYDI data. The main objectives were (i) to describe the epidemiological situation of LB in Bavaria, (ii) to assess clinical manifestations and treatment of LB, and (iii) to identify areas and populations with an increased risk for LB. Our results aim to serve as a basis for developing tailored strategies to prevent LB infection and increase awareness of LB among physicians and the general population.

## 2. Materials and Methods

### 2.1. Data Sources

Two data sources were used in this study to examine the epidemiological situation of LB in Bavaria. First, we analyzed data collected via the mandatory notification of LB in Bavaria: Physicians (both general practitioners (GPs) and those working in hospitals) report anonymized LB cases to the local heath authority; notifications are then transmitted electronically to the Bavarian Health and Food Safety Authority (LGL). We included LB cases that were reported between 1 March 2013 and 31 December 2020. Additionally, we used TBE data reported in the same period. Second, we analyzed data acquired through LYDI, a physician-based LB sentinel network, from Bavaria between 2013 and 2016 [22]. Participating practitioners were offered education by LB symposia and by providing relevant literature (guidelines, expert opinions), and they could discuss individual cases by phone or e-mail with experts from the German National Reference Center for *Borrelia*.

### 2.2. Definition of Variables

A notifiable case of LB is defined as a person who has any of the three following manifestations: EM, acute NB, or LA. The case definitions for the respective manifestations are provided by the Bavarian state health authority LGL (https://www.lgl.bayern.de/downloads/gesundheit/infektionsschutz/doc/falldefinition_lyme_borreliose.pdf, accessed on 2 September 2021). EM is clinically diagnosed as a progressively expanding red or bluish-red rash or several rashes, which are often circular or ring-shaped with gradual clearing originating from the center. Acute NB is clinically diagnosed as acute, painful radiculoneuritis, neuritis cranialis, or meningitis. In addition to these clinical criteria, the diagnosis of NB requires laboratory confirmation via detection of the presence of lymphocytic pleocytosis in the cerebrospinal fluid (only necessary for radiculoneuritis/meningitis), as well as at least one of the following: (i) intrathecally produced specific antibodies, (ii) nucleic acids of *B. burgdorferi* s.l. (i.e., positive PCR), (iii) *B. burgdorferi* s.l. in culture, or (iv) serum anti *B. burgdorferi* immunoglobulin (Ig) G antibodies (only sufficient for neuritis cranialis and for persons ≤18 years). LA is clinically diagnosed as mono-/ oligoarthritis of large joints excluding arthritis of other causes. Additionally, LA needs to be laboratory-confirmed by the presence of (i) IgG antibodies in serum, (ii) nucleic acids of *B. burgdorferi* s.l. in joint punctate (i.e., positive PCR), or (iii) *B. burgdorferi* s.l. in culture taken from joint punctate. All cases analyzed here met the case definition mentioned above.

### 2.3. Statistical Analysis

Data were analyzed using STATA12 (StataCorp LP, College Station, TX, USA). Incidences were calculated per 100,000 inhabitants based on population estimates of the Bavarian State Office for Statistics (https://www.statistikdaten.bayern.de/genesis/online, accessed on 6 January 2021). *p*-values < 0.05 were considered statistically significant. We conducted univariable logistic regression analyses to determine associations of age and sex with clinical manifestations. The incidence map was produced using RegioGraph (GfK GeoMarketing) and shows the administrative districts in Bavaria. Data query (notification data) for this study was performed on 1 January 2021.

### 2.4. LYDI Sentinel Network

The LYDI sentinel network involved cooperation with GPs, dermatologists, rheumatologists, and neurologists in Bavaria. Data on symptoms, laboratory diagnostics and therapy were obtained by the physicians via questionnaires. Pseudonymized data were then transferred to the LGL. Therapeutic success was obtained from participants via participating physicians after completion of the sentinel network. For the definition of the manifestations EM, LA and NB, we used the same criteria as for LB notification (see above). Persons aged 18 years or above who met the case definition were included in the LYDI sentinel network.

### 2.5. Ethics

The LYDI sentinel was approved by the Ethics Committee of the Bavarian Medical Chamber (no: 12006; date of last valid version: 30 April 2014). All patients included in the LYDI-sentinel signed an informed consent form and thus agreed to the publication of their data. Information of individual patients was transferred to LGL in a pseudonymized form. At no time did the LGL have information on the identity of the patients. Name and contact information were only known to the attending physician/hospital. The LYDI-sentinel was financed by means of the Bavarian Ministry of Health and Care.

## 3. Results

### 3.1. Mandatory Notification

#### 3.1.1. Burden of Disease

Of 36,219 reported LB cases, 35,458 (98%) fulfilled the criteria for acute disease and were included in further analyses (Table 1). Average annual incidence was 34.3 cases per 100,000 inhabitants during 2013–2020. Annual incidence ranged from 23.2 cases/100,000 in 2015 to 47.4 cases/100,000 in 2020.

#### 3.1.2. Demographic Aspects

**Figure 1:**
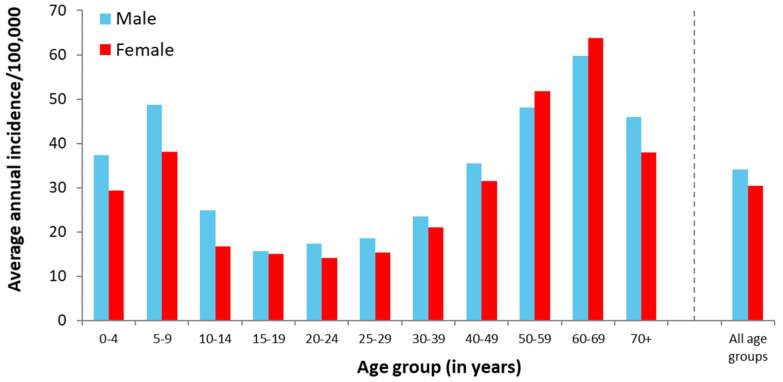
Average annual incidence (reported cases per 100,000 inhabitants) of LB in Bavaria, Germany, during 2013–2020 by age group and sex (*n*=34,002 with information on age and sex available).

Acute LB occurred slightly more frequently in females (53.8%; 18,288 of 34,002 reported cases with information available) compared to males. Overall, but especially among children and adolescents up to the age of 14 years, incidence was higher in males (Figure 1). The age-specific incidence of LB showed a two-peak distribution (Figure 1). The first peak corresponds to the age group 5–9 years, and the second to the age group 60–69 years. While in the first peak incidence was higher in males, it was slightly higher in females in the second peak (Figure 1).

#### 3.1.3. Seasonality

**Figure 2:**
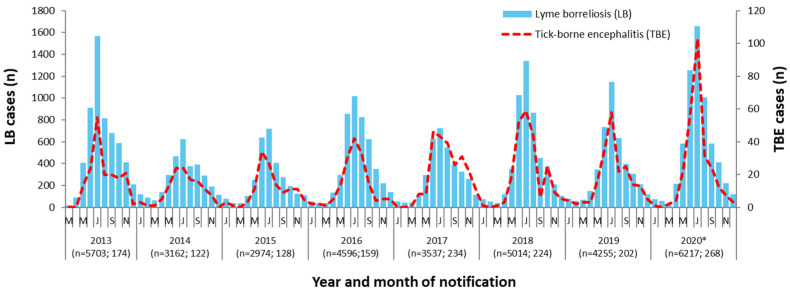
Reported cases of LB (*n* = 35,458) and tick-borne encephalitis (*n* = 1511) in Bavaria during the first 8 years of mandatory LB notification (March 2013–December 2020), by year and month of notification; numbers below year dates indicate annual notified LB and TBE cases, respectively; * the 2020 season was likely influenced by the COVID-19 pandemic and periods of lockdown (see discussion section).

During the first 8 years of routing surveillance in Bavaria, seasonal occurrence of LB cases was similar between notification years (Figure 2): The majority of LB cases (58.6%) occurred during the meteorological summer (June–August), with a further 11.9% and 24.0% during spring (March–May) and autumn (September–November), respectively, and the remaining 5.5% during the winter months (December–February). Nonetheless, we observed marked differences in the total number of notified cases between individual years (range: 2974–6217 cases per year). When comparing LB case numbers with those of TBE, the second most common tick-borne disease in Germany, it is apparent that both the temporal occurrence as well as the number of cases correlate each season between the two tick-borne diseases (Figure 2).

#### 3.1.4. Clinical Aspects

Of 35,458 LB cases included in the study, 34,283 (96.7%) were reported to have EM as a manifestation (Table 1). In total, 615/35,458 (1.7%) cases fulfilled the criteria for NB and 633/35,458 (1.8%) for LA (multiple manifestations were possible). Among NB cases, the most frequently reported manifestation was neuritis cranialis (367/615; 59.7% of NB cases), followed by radiculoneuritis (246/615; 40.0%) and meningitis (164/615; 26.7%).

**Figure 3:**
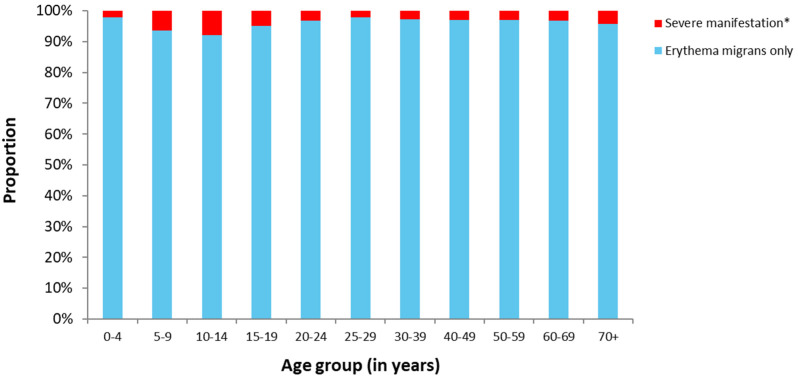
Proportion of LB cases reported between 2013 and 2020 in Bavaria, Germany, by severity of manifestation and age group (*n* = 34,840 with information on age available; * Severe manifestations were defined as reported LA, radiculoneuritis, meningitis, and neuritis cranialis).

No LB-attributable deaths were reported between 2013 and 2020 in Bavaria. The proportion of cases with any severe manifestation of LB (NB or LA) was lowest in 25–29-year-olds (2.1%) and varied from 2.2% to 7.9% in the remaining age groups (Figure 3).

Univariable analysis showed that children aged 5–9 years had significantly increased odds of developing meningitis (odds ratio (OR): 3.4; 95% confidence interval (CI): 2.2–5.2; Table 2) as well as neuritis cranialis (OR: 4.6; 95% CI: 3.6–6.0) compared to all other age groups. Apart from meningitis, males had significantly increased odds of developing all the severe LB manifestations considered in this study (Table 2).

Information on hospitalization status was available for 28,669/35,458 (80.9%) cases. Whereas patients with EM and LA were hospitalized in only 0.8% and 6.6% of cases, respectively, the hospitalization rate was relatively high among NB cases (85.6%). Among those cases with information available (29,273/35,458), 16,388 (56.0%) recalled a tick bite prior to LB infection. Information on the *B. burgdorferi* species was available for 8.0% (2,830/35,458) of reported LB cases. Of these, 2,599 (91.8%) were classified as *B. burgdorferi* sensu lato. Among cases with further specification of *B. burgdorferi* isolates (231/2830), the most common species found was *B. burgdorferi* s.s. (115/231; 49.8%), followed by *B. afzelli* (87/231; 37.7%), *B. garinii* (13/231; 5.6%), *B. bavariensis* (9/231; 3.9%), and *B. spielmanii* (7/231; 3.0%).

#### 3.1.5. Geographic Aspects/Risk Areas

**Figure 4:**
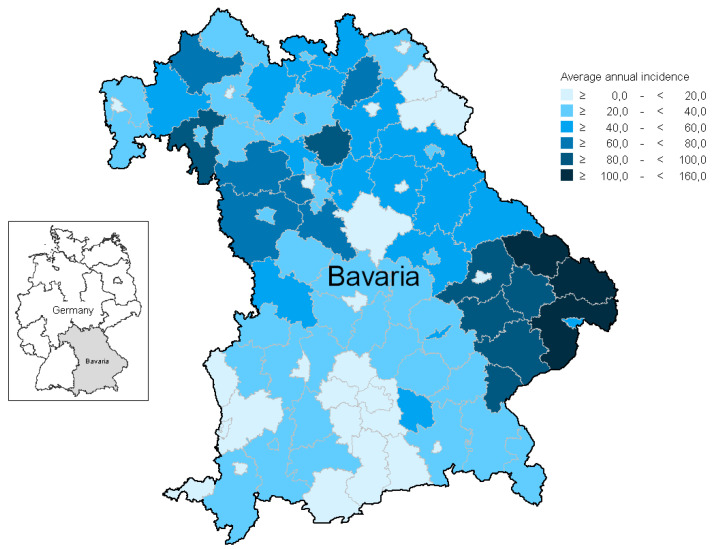
Average annual LB incidence (notified cases per 100,000 inhabitants) in Bavaria, Germany, 2013–2020, by district.

Average annual LB incidence by administrative district is shown in Figure 4. Incidence varied substantially between districts (range: 4–154 cases/100,000 inhabitants; interquartile range (IQR): 20–52) and was highest in districts belonging to the Bavarian Forest region, located in the border area between Germany, Austria, and the Czech Republic. Further high-incidence districts (>70 cases/100,000) were identified in the Franconia region in northern and northwestern Bavaria (Figure 4).

### 3.2. LYDI Sentinel Network

In total, 284 medical practices (121 general practitioners, 67 neurological, 74 dermatological, 22 rheumatological practices) as well as four neurological hospital wards participated in the LYDI sentinel network. During the study period (2013–2016) they reported 282 cases that met the inclusion criteria for acute LB. The distribution of clinical manifestations among LYDI patients was similar to that observed in mandatory notification data. In the LYDI sentinel network, 97.2% showed an EM, 0.4% NB, and 3.2% LA. Approximately 45% of patients recalled a tick bite prior to LB infection. One in three patients (36%) reported having had accompanying symptoms like fatigue, joint or muscle aches, headache, and fever. In 29% of cases, the EM was between 5 and 9 cm in diameter, in a further 29% between 10 and 14 cm, and in 16% between 15 and 19 cm in diameter. The remaining 26% of cases did not visit a physician until the EM had a diameter of 20 cm or larger. No considerable difference was found regarding EM localization with respect to the transversal axis (occurrence on the right side 52%; left side 48%). Generally, the EM was found more frequently on the ventral part of the body (61%) than the dorsal part (39%). The median incubation period (date of tick bite until symptom onset) was 10 days (range: 0–124 days; IQR: 2–20 days). Overall, 277 of 282 patients (98%) were treated with antibiotics, 85% of which were treated in accordance with official guidelines regarding duration and dosage of antibiotic treatment. Doxycycline was used in 91% of treated patients, amoxicillin in 6%, cefuroxime in 2%, and rocephin in 1%. Of 277 patients treated with antibiotics, 244 (88%) gave feedback by questionnaire directly after the end of the study regarding the therapeutic success, which was 100%.

## 4. Discussion

A prerequisite for the local LB risk assessment and implementation of targeted preventive measures is an understanding of the burden of disease in the population as well as of the demographic and regional distribution of LB. Analyzing data obtained from more than 35,000 LB cases reported during the first 8 years of routine surveillance as well as from a 3-year physician-based sentinel network, our study provides a comprehensive overview of the epidemiological situation of LB in Bavaria, Germany.

### 4.1. Regional Distribution of LB

In accordance with other German studies based on LB surveillance data [24,25], the regional distribution of LB cases showed substantial heterogeneity (Figure 4). We identified both the Bavarian Forest region in eastern Bavaria, as well as parts of upper, lower and central Franconia in northwest Bavaria as areas with an increased risk of contracting LB. Since several recreational areas are located in the identified regions, it can be assumed that people living there spend more time outdoors and are therefore more frequently exposed to ticks and hence also to *Borrelia*. Nonetheless, it remains unclear whether additional factors, such as the proportion of ticks infected with *B. burgdorferi* or the general tick density, contribute to this phenomenon in the identified areas. This needs to be investigated in further studies.

### 4.2. Demographic Aspects

As also described previously in several national and international studies (e.g., [24,25,26,27,28]), we observed a bimodal age distribution in our study. Consistent with the findings of for example, Enkelmann et al. [24] in a study that included some of the same data we analyzed from 2013 to 2017, or Wilking et al. [25] and Fülöp et al. [26], the first incidence peak occurred among 5–9-year-old males; the second peak was observed in the group aged 60–69 years. A similar bimodal age distribution has also been reported for other countries, e.g., the United States and France, but while in France the second peak occurs in those aged 70–79 years, it occurs among 50–55-year-olds in the United States [27,28]. Comparable to the above-mentioned German studies [24,25,26], incidence in the older age group was considerably higher in females; we observed this only for the years 2013–2019 in our study (Supplementary Figures S1–S8). In 2020, however, our results instead rather resemble the findings of population-based seroprevalence studies conducted in Germany, which reported significantly higher LB antibody prevalence in male individuals across all age groups [4,5]. Previous studies have hypothesized that adult males in Germany might be less likely to seek medical advice when detecting an EM compared to women [24,25]. Our findings for 2020 may indicate a change in health-seeking behavior during the pandemic, meaning that women with EM might have avoided seeking medical attention for EM due to fear of contracting COVID-19. On the other hand, the willingness of men to present themselves to a physician at all when detecting an EM may have increased due to the influence of the pandemic. The latter is supported by the fact that both the number of reported cases and the proportion of EMs were higher in 2020 compared with the other years.

In general, both the higher incidence in males and the two age-dependent incidence peaks might be explained by differences in outdoor behavior between males and females and between the different age groups. Both children aged 5–9 years and persons at the beginning of retirement age presumably spend more time in nature and are more often exposed to ticks (and thus also to *Borrelia*).

### 4.3. Clinical Aspects

EM was reported for a large proportion of notified cases in our study. The results of the LYDI study show that more than one-third of cases did not seek medical attention until the EM exceeded 15 cm in diameter. For a favorable prognosis of LB, however, it is important to diagnose LB early and treat it adequately with antibiotics. Accordingly, the population should be sensitized to seek medical help right away at the onset of EM.

With just under 2% each, the proportion of the disseminated manifestations NB and LA was low among reported LB cases in our study. Considerably higher proportions of NB were reported in prospective studies from other countries, such as Sweden at 16% [29] and the United States at 12.5% [30]. Compared to our findings, a markedly higher proportion (27.5%) was also described for LA in the United States, but LB cases in the United States are mainly caused by *B. burgdorferi* s.s., a species known to cause LA. Similar to the details presented in the limitations below and also described by Enkelmann et al. [24], the low proportion could be in part due to the complex case definition of NB and LA in Germany. In the study period, a total of 250 cases with symptoms of NB (radiculoneuritis, meningitis, neuritis cranialis) and 330 cases with a reported LA were not included in the analysis due to the lack of additional information to completely fulfil the case definition.

According to the results presented here, children aged 5–14 years generally had a higher risk of developing a severe form of LB. Therefore, our findings are in line with previous research reporting a higher risk for meningitis and peripheral facial palsy in children compared to adults [13].

### 4.4. Seasonality

The occurrence of LB in Bavaria is subject to pronounced seasonality, with most cases being reported in the summer months. This corresponds with the tick’s host-seeking behavior as well as the population’s increased recreational activity outdoors. The total number of reported cases did, however, vary substantially between the years analyzed in this study. These differences are probably due to the complex interaction of various factors. On the one hand, the differences could be explained by weather-related tick activity and the changing outdoor behavior of the population depending on the weather. On the other hand, tick density varies from year to year, as does the proportion of ticks infected with *Borrelia*. Other climatic and ecological factors may also play a role and have been previously discussed in several reports and studies [31,32,33]. In our study, we additionally showed that seasonal variations in LB correlate strongly with those of TBE, the second most common tick-borne disease. The complex interplay of the aforementioned factors and its effect on seasonal variability in the incidence of tick-borne diseases should be investigated in detail in further studies.

### 4.5. LB in Times of COVID-19

In 2020—the first year of the COVID-19 pandemic in Germany—more LB cases than ever before since the implementation of mandatory notification in Bavaria were reported. At the same time, the proportion of severe LB manifestations (LA and NB) was substantially lower compared to previous years. High case numbers during 2020 may be due to several reasons. First, a high tick density was observed in 2020 [34]. Moreover, as a result of the lockdown in the spring and summer of 2020 that, for example, forced parents to care for their children at home, led to part-time work or unemployment for many, resulted in limited options for indoor sports, and prevented people from spending their vacation time in popular holiday destinations, many people in Bavaria may have spent more time closer to their home in nature. Consequently, people were exposed to ticks and were presumably bitten more frequently, therefore having a higher risk of becoming infected. Additionally, the weather in spring and early summer 2020 was nice and warm in Bavaria. This may also have led to people spending more time outdoors. However, it remains unclear why the proportion of severe disease manifestations was lower in 2020. It is conceivable that due to the pandemic, fewer people with LA or NB consulted a doctor and therefore severe LB manifestations may have been underdiagnosed. The negative impact of the COVID-19 pandemic on the timely diagnosis of tick-borne infections was also described by Wormser and colleagues [35]. An alternative explanation for this phenomenon would be that those affected by (usually less symptomatic) EM had more time to see a doctor due to the external circumstances during the pandemic. As a result, EM would have been diagnosed and reported more frequently. A further explanation could be that people still had good access to their GP via video consultations, which would have allowed the diagnosis of an EM. This is supported by the fact that the number of teleconsultations in Germany increased from 3000 in 2019 to 1.4 million just in the first 6 months of 2020 [36]. People with a NB or LA may have presented themselves at a hospital, but—in absence of a real emergency situation compared to COVID-19—may have received antibiotic treatment for a possible LB without further diagnostic clarification. In addition, it might be possible that due to the immense workload at local health authorities caused by COVID-19, the—often complex and time-consuming—investigations necessary for LA and NB cases were postponed. This might have led to a lower number of reported cases with severe LB manifestations meeting the case definition.

### 4.6. Strengths and Limitations

One strength of our report is that our findings were based on many reported LB cases (>35,000). Being able to complement data from mandatory LB notification with data acquired via the LYDI sentinel network (e.g., on clinical aspects) gives a more comprehensive overview of the epidemiological situation of LB and was therefore a further asset. However, our study was prone to some limitations that should be acknowledged. In general, a high level of underreporting can be assumed regarding LB notification. According to estimates of the US CDC, the actual number of LB cases is 10-fold higher than the number of reported cases [37]. Due to the anonymous reporting obligation in Bavaria—meaning that neither the name nor the full date of birth are reported to the local health authorities—it is difficult to trace back individual cases. In addition, case definitions for NB and LA are complex and therefore prone to incomplete or incorrect data entry. Together with the limited possibility to trace back cases, this might have led to further underreporting, especially of NB and LA cases. Despite the fact that this approach might have led to a less precise depiction, we used the variable county of residency (information available for 100% of notified cases) instead of the place (county) where the infection was most likely acquired (available only for 65%) to analyze the regional distribution of LB in our study. However, since Enkelmann et al. found that >90% of reported cases acquired the LB infection in the county of residency [18], we do not believe that this led to any major distortions. Moreover, only 10 months could be analyzed for the notification year 2013 due to the fact that mandatory notification started in March. Since only approximately 2% of LB cases are notified in the missing months of January and February, we consider this negligible.

## 5. Conclusions

The risk of acquiring LB in Bavaria, Germany, is substantial. Areas in Bavaria with a particularly high risk for LB included the Bavarian Forest as well as parts of Franconia. An increased risk was also identified for boys aged 5–9 years as well as female persons aged 60–69 years. In contrast to TBE, there is currently no vaccination available against LB. Measures to prevent LB should therefore aim to avoid tick bites, e.g., by wearing long, light-colored clothing, using repellents, and thoroughly checking for ticks after spending time outdoors. To minimize the risk of transmitting *Borrelia* to humans, ticks should be removed as soon as possible after the bite. The general population should hence be sensitized to these issues. For a favorable prognosis of LB, it is additionally important to initiate therapy with adequate antibiotics at an early stage of infection. The awareness of both the population as well general physicians in this regard should further be increased, e.g., through targeted information campaigns.

## Figures and Tables

**Table 1:** Reported cases and clinical manifestations of LB in Bavaria, Germany, 2013–2020 (*n* = 35,458).

Diagnosis	2013 (Mar–Dec)		2014		2015		2016		2017		2018		2019		2020		2013–2020	
	*n*	%	*n*	%	*n*	%	*n*	%	*n*	%	*n*	%	*n*	%	*n*	%	*n*	%
Lyme borreliosis (LB)	5703	100	3162	100	2974	100	4596	100	3537	100	5014	100	4255	100	6217	100	35,458	100
Erythema migrans (EM)	5593	98.1 ^#^	2980	94.2 ^#^	2,825	95.0 ^#^	4395	95.6 ^#^	3393	95.9 ^#^	4869	97.1 ^#^	4120	96.8 ^#^	6108	98.2 ^#^	34,283	96.7
Acute neuroborreliosis (NB) *thereof*	64	1.1 ^#^	66	2.1 ^#^	71	2.4 ^#^	101	2.2^#^	68	1.9 ^#^	81	1.6 ^#^	82	1.9 ^#^	82	1.3 ^#^	615	1.7
Radiculoneuritis	29	45.3 *	18	27.3 *	31	43.7 *	37	36.6 *	21	30.9 *	43	53.1 *	40	48.8 *	27	32.9 *	246	40.0
Meningitis	15	23.4 *	16	24.2 *	21	29.6 *	31	30.7 *	15	22.1 *	15	18.5 *	21	25.6 *	30	36.6 *	164	26.7
Neuritis cranialis	34	53.1 *	45	68.2 *	42	59.2 *	58	57.4 *	44	64.7 *	50	61.7 *	45	54.9 *	49	59.8 *	367	59.7
Lyme arthritis (LA)	50	0.9 ^#^	125	4.0 ^#^	87	2.9 ^#^	111	2.4 ^#^	84	2.4 ^#^	75	1.5 ^#^	69	1.6 ^#^	32	0.5 ^#^	633	1.8
EM and NB ^+^	2	-	4	-	7	-	6	-	3	-	7	-	12	-	4	-	45	-
EM and LA ^+^	2	-	5	-	2	-	5	-	5	-	4	-	4	-	1	-	28	-

Mar = March; Dec = December; ^#^ % of Lyme borreliosis cases; * % of neuroborreliosis cases; ^+^ multiple diagnoses included above.

**Table 2:** Association of age and sex with clinical manifestations among LB cases (*n* = 35,458) reported from 2013 to 2020 in Bavaria, Germany: Results of univariate logistic regression analysis.

	Lyme Arthritis OR (95% CI)	Radiculoneuritis OR (95% CI)	Meningitis OR (95% CI)	Neuritis Cranialis OR (95% CI)
**Sex**				
Female	Ref.	Ref.	Ref.	Ref.
Male	1.36 (1.16–1.60) **	1.84 (1.42–2.38) **	1.00 (0.73–1.37)	2.07 (1.67–2.57) **
**Age group 1**				
All other age groups	Ref.	Ref.	Ref.	Ref.
5–9 years	0.83 (0.56–1.21)	0.15 (0.04–0.58) **	3.40 (2.23–5.18) **	4.63 (3.58–6.01) **
**Age group 2**				
All other age groups	Ref.	Ref.	Ref.	Ref.
Age 60–69 years	0.88 (0.72–1.08)	1.62 (1.22–2.14) *	0.71 (0.46–1.09)	0.63 (0.47–0.85) *

OR = Odds Ratio; CI = confidence interval; Ref. = reference group; * *p* < 0.01; ** *p* < 0.001.

## Data Availability

The data presented in this study are available on request from the corresponding author. The data are not publicly available due to statutory obligations.

## Supplementary Materials

The following are available online at https://www.mdpi.com/article/10.3390/microorganisms9091872/s1. Figures S1–S8: Annual incidence (reported cases per 100,000 inhabitants) of LB in Bavaria, Germany, years 2013 (S1) to 2020 (S8) by age group and sex.
