# Supplementary material for: Epidemiological Surveillance of Lyme Borreliosis in Bavaria, Germany, 2013–2020
Source: Microorganisms. 2021 Sep 3;9(9):1872. doi: 10.3390/microorganisms9091872 (PMC8467410; doi:10.3390/microorganisms9091872)

# Supplement

Böhmer MM, Ens K, Böhm S, Heinzinger S, Fingerle V: **Epidemiological Surveillance of Lyme Borreliosis in Bavaria, Germany, 2013-2020**. Microorganisms 2021.

---

**Figure S1**

Annual incidence (reported cases per 100,000 inhabitants) of Lyme borreliosis in Bavaria, Germany, 2013 by age group and sex (*n*=5,414 with information on age and sex available)

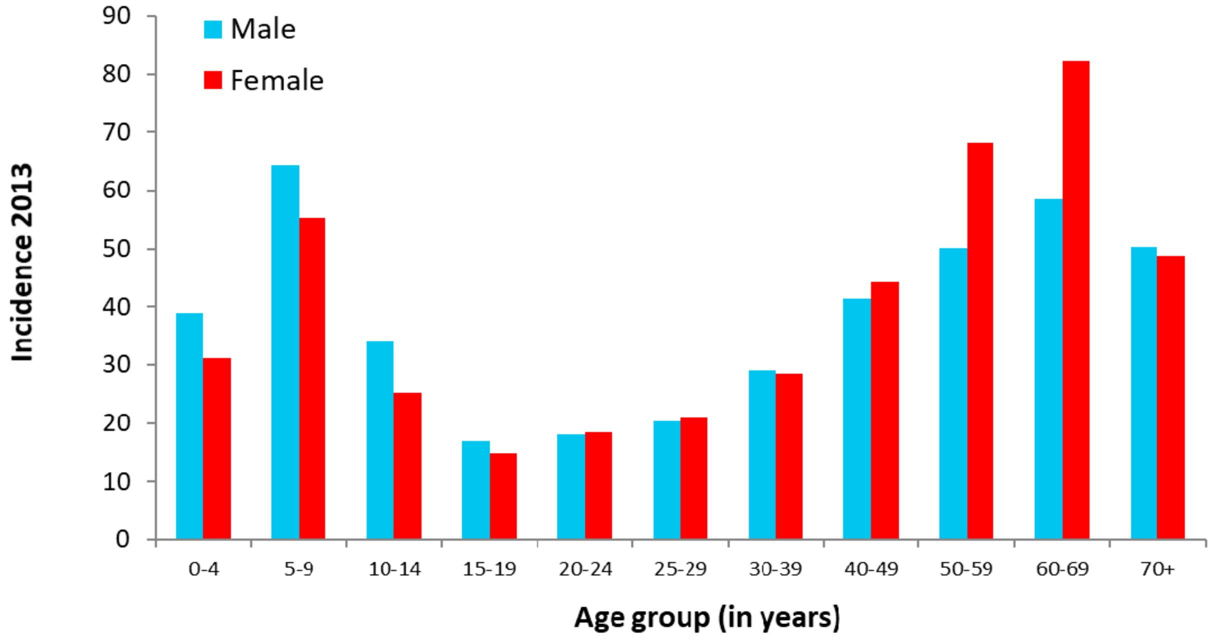

**Figure S2**
Annual incidence (reported cases per 100,000 inhabitants) of Lyme borreliosis in Bavaria, Germany, 2014 by age group and sex (*n*=3021 with information on age and sex available)

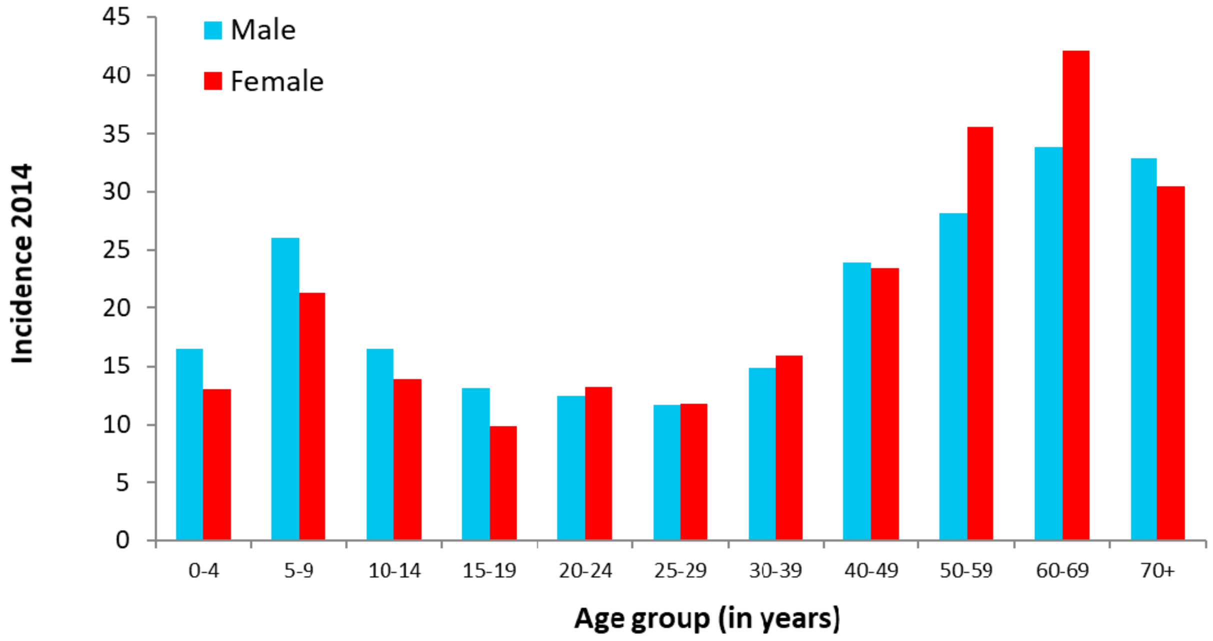


**Figure S3**
Annual incidence (reported cases per 100,000 inhabitants) of Lyme borreliosis in Bavaria, Germany, 2015 by age group and sex (*n*=2858 with information on age and sex available)

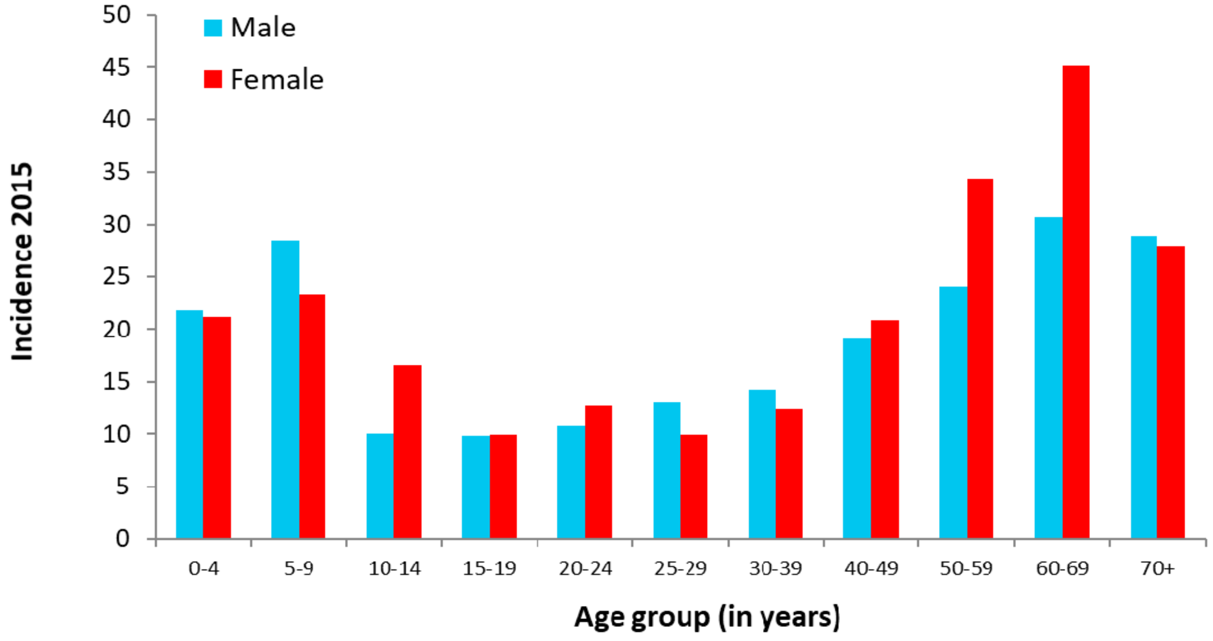

**Figure S4**
Annual incidence (reported cases per 100,000 inhabitants) of Lyme borreliosis in Bavaria, Germany, 2016 by age group and sex (*n*=4,443 with information on age and sex available)

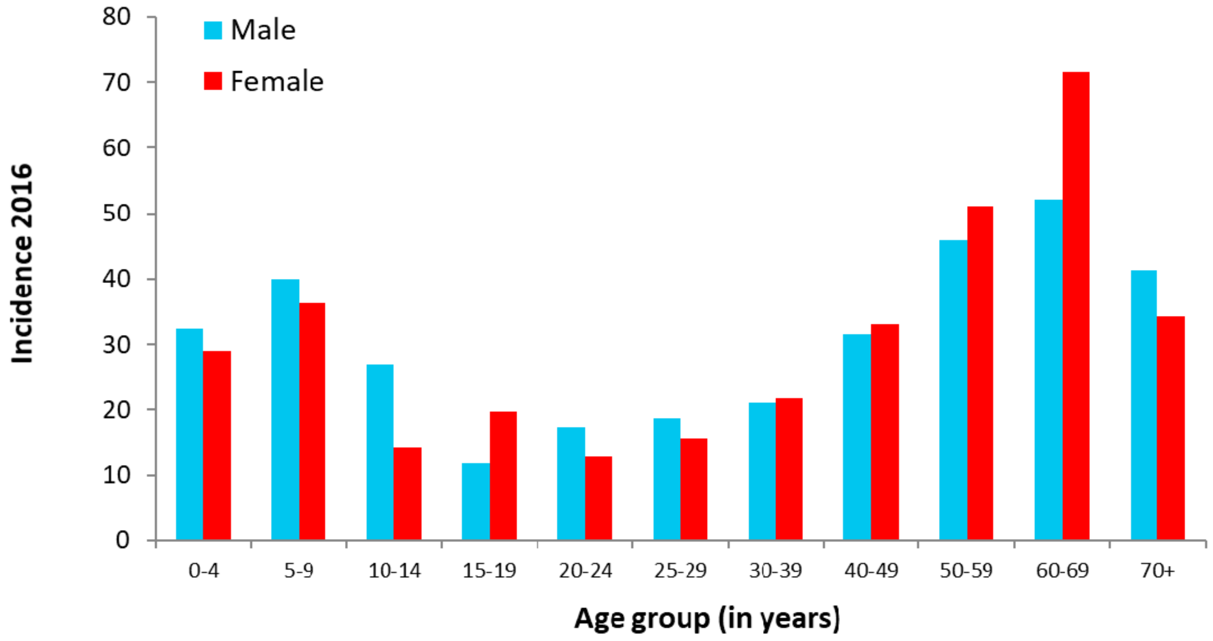


**Figure S5**
Annual incidence (reported cases per 100,000 inhabitants) of Lyme borreliosis in Bavaria, Germany, 2017 by age group and sex (*n*=3,394 with information on age and sex available)

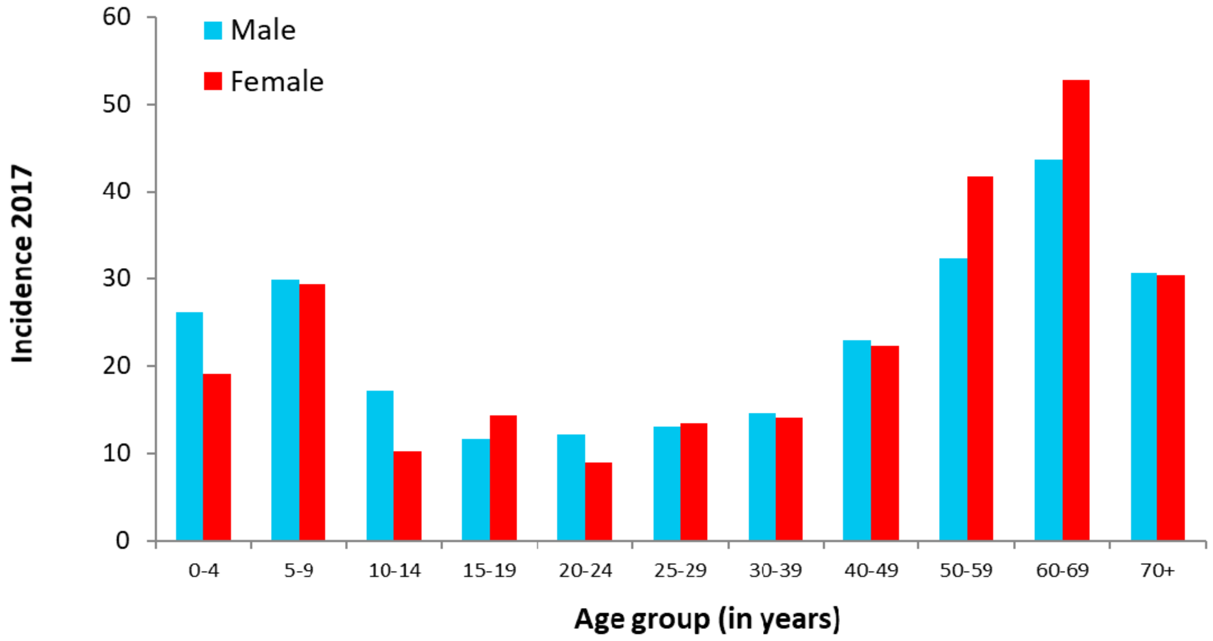

**Figure S6**

Annual incidence (reported cases per 100,000 inhabitants) of Lyme borreliosis in Bavaria, Germany, 2018 by age group and sex (*n*=4,797 with information on age and sex available)

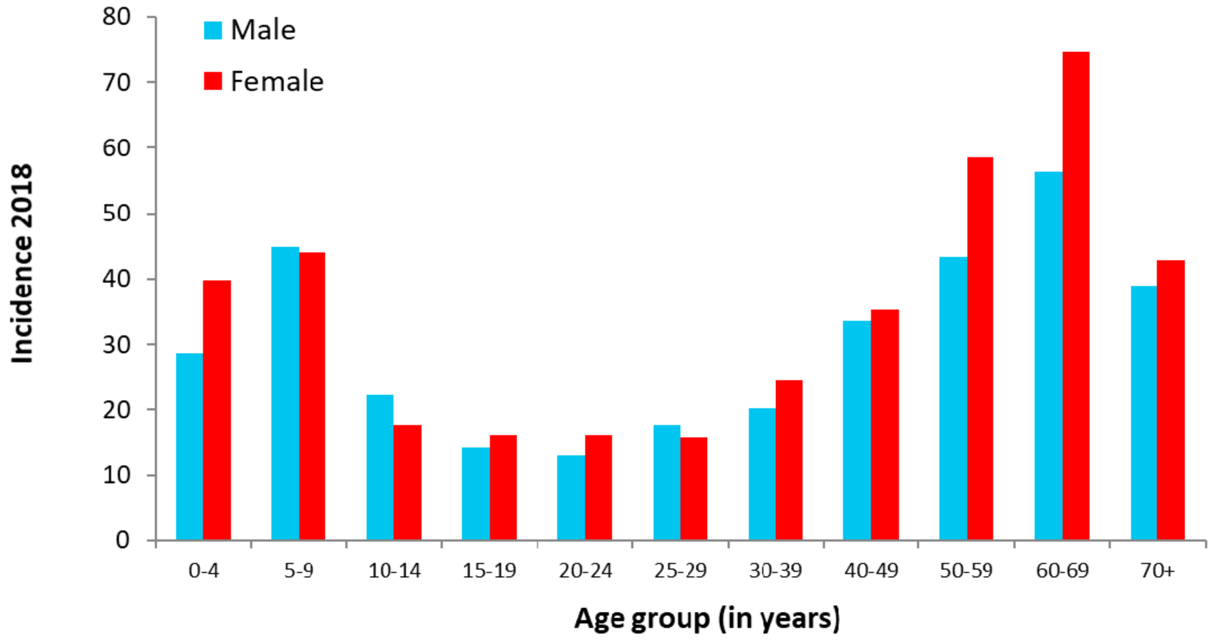


**Figure S7**

Annual incidence (reported cases per 100,000 inhabitants) of Lyme borreliosis in Bavaria, Germany, 2019 by age group and sex (*n*=4,068 with information on age and sex available)

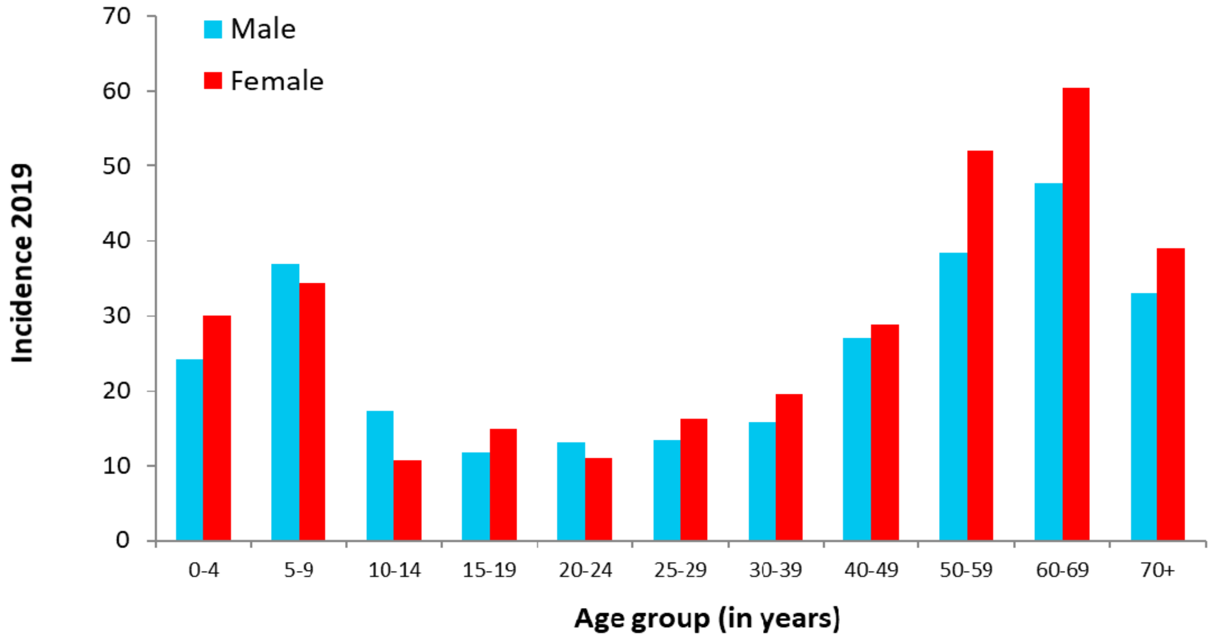

**Figure S8**

Annual incidence (reported cases per 100,000 inhabitants) of Lyme borreliosis in Bavaria, Germany, 2020 by age group and sex (*n*=6,007 with information on age and sex available)

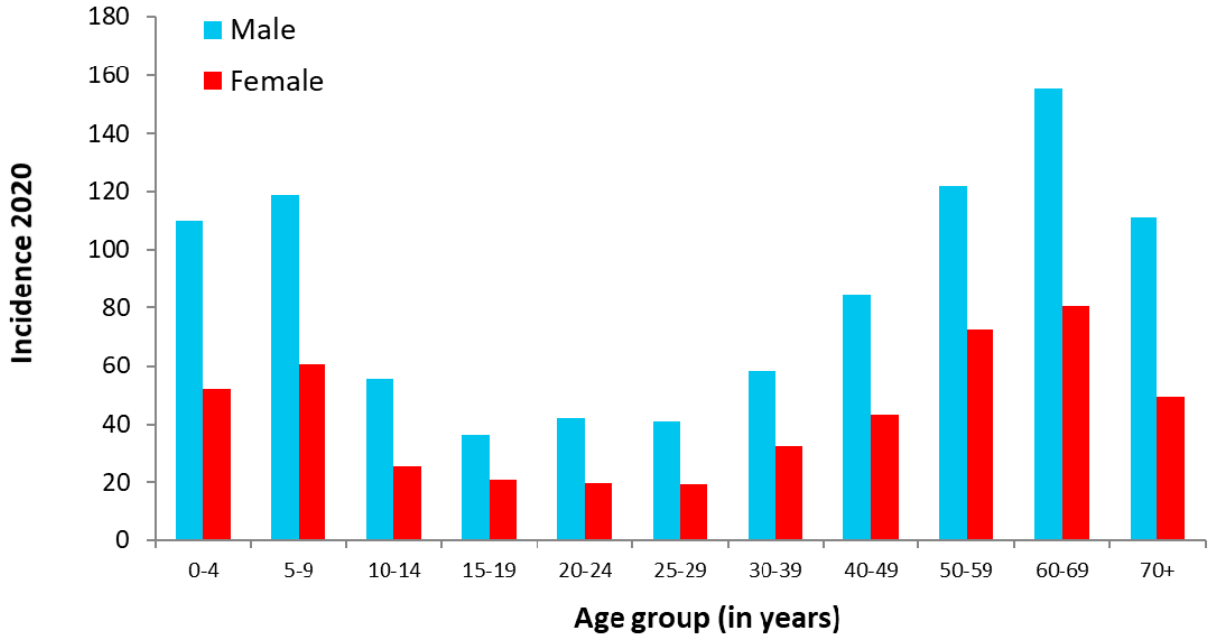

Supplement: Supplementary file 1 [file microorganisms-09-01872-s001.zip › microorganisms-1336165-supplementary.pdf]
